# Towards Rabies Elimination in Pakistan: Barriers, Facilitators, and the Role of One Health

**DOI:** 10.1007/s44197-025-00441-7

**Published:** 2025-08-06

**Authors:** Anum Shaikh, Shifa Salman Habib, Ali Faisal Saleem, Naseem Salahuddin

**Affiliations:** 1 Department of Community Health Sciences, Aga Khan University, Karachi, Pakistan; 2 Department of Pediatrics and Child Health, Aga Khan University, Karachi, Pakistan; 3 Department of Infectious Diseases, Indus Hospital & Health Network (IHHN), Karachi, Pakistan

**Keywords:** Rabies control, Zoonotic Diseases, One Health, Mass dog vaccination, TNVR, Pakistan

## Abstract

**Background:**

Rabies is a neglected zoonotic disease with an estimated 59,000 deaths annually, disproportionately affecting low- and middle-income countries. Pakistan remains a high-burden setting due to weak surveillance, poor intersectoral coordination, and limited public awareness. Therefore, this study aimed to examine the barriers and facilitators to rabies control in Pakistan through the One Health approach, integrating perspectives from both community members and institutional stakeholders.

**Methods:**

This mixed-methods study design was conducted in Karachi, where 385 household respondents completed a structured Knowledge, Attitudes, and Practices (KAP) survey, and 10 stakeholders (out of 14 approached) were interviewed across human, animal, and environmental sectors. The survey tool was adapted from previously validated instruments and pilot-tested. Thematic analysis was conducted using a deductive framework based on One Health principles. The quantitative data were analyzed descriptively using IBM SPSS Statistics 21, whereas the qualitative data were analyzed using Atlas.ti.

**Results:**

The average knowledge score was 5.54 out of 13 (42.6%), indicating substantial knowledge gaps among community members. Key barriers identified included limited vaccine availability, inadequate surveillance systems, fragmented dog population control, and weak multisectoral collaboration. Enabling factors included stakeholder willingness, local Trap-Neuter-Vaccinate-Release (TNVR) initiatives, and existing collaborative frameworks. Stakeholder awareness of the One Health approach was present but lacked institutional translation.

**Conclusion:**

A coordinated, One Health-based strategy for rabies elimination is urgently needed in Pakistan that addresses both systemic and community-level gaps through sustained advocacy, stronger intersectoral coordination, expansion of TNVR initiatives, and establishment of centralized surveillance for bite incidents and post-exposure management.

## Background

Rabies is a fatal viral zoonosis responsible for an estimated 60,000 human deaths annually, with the highest burden concentrated in Asia (59.6%) and Africa (36.4%), where dog-mediated transmission accounts for the vast majority of the cases [1, 2]. The disease continues to disproportionately affect low- and middle-income countries, where access to post-exposure prophylaxis and sustainable canine vaccination remains limited [1].

In South Asia, estimates from 2015 placed India, Pakistan, and Bangladesh among the top five countries with rabies burden, accounting for 25,000 deaths annually, and exposing over 1.4 billion people to the risk [3, 4]. Within this context, Pakistan currently reports approximately 1,037 rabies-related deaths annually [5], reflecting a persistently high mortality rate despite the preventability of the disease.

On average, government hospitals in Pakistan receive 50–70 dog bite cases per day [6]. The burden is particularly evident in Karachi, which alone reports 25–30 bite cases daily at a tertiary care hospital [7, 8]. Recent estimates place Karachi’s annual rabies incidence between 7 and 9.8 cases per million population [8, 9]. The city’s large stray dog population has made it a pilot site for TNVR campaigns led by municipal authorities and civil society partners [10–12]. Despite the burden, rabies continues to be under-prioritized in Pakistan’s national health agenda due to weak surveillance, fragmented implementation of dog population control laws, and limited intersectoral accountability [13–15].

**Fig. 1:**
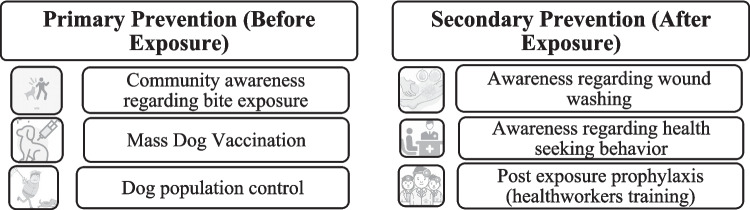
Primary and secondary prevention of rabies according to the One Health approach [17]

Globally, the One Health approach − integrating human, animal, and environmental health perspectives − has proven effective in rabies control and prevention, especially in resource-limited settings (see Fig. 1) [16, 17]. Recent literature emphasizes that successful implementation also depends on the legal context, social structures, and economic factors that shape policy and practice [18, 19]. For complex zoonosis like rabies, these broader determinants are integral to effective, sustainable control.

Following the implementation of multisectoral strategies, countries like China and Bhutan reported notable declines in rabies mortality [16, 20]. Bangladesh, which was previously listed among the high-burden countries, also achieved a substantial decline in rabies-related mortalities through coordinated One Health-based intervention and strong political commitment [21]. India has also initiated its National Rabies Control Program (NRCP) in 2023 based on the One Health [22]. In contrast, One Health remains underutilized in Pakistan, with no formal institutional mechanisms or cohesive policies to guide intersectoral coordination.

Although rabies control initiatives such as dog culling, sporadic TNVR campaigns, and post-exposure prophylaxis availability exist, they are largely siloed, lacking integration between veterinary, public health, and municipal agencies. Furthermore, empirical studies using a One Health lens to examine rabies control in Pakistan remain limited, underscoring the need for evidence-informed strategies that align with the country’s decentralized health governance and urban challenges. This study, therefore, seeks to address this gap by exploring the barriers and facilitators to rabies control and elimination in Karachi, Pakistan, and examining how the One Health approach can be effectively operationalized to address these barriers.

## Methods

### Study Aims and Objectives

This study aimed to identify the barriers and facilitators to rabies control in Pakistan through a One Health approach, by integrating community perspectives and stakeholder insights across the human, animal, and environmental health sectors to inform context-specific, multi-sectoral rabies control strategies.

### Conceptual Framework

**Fig. 2:**
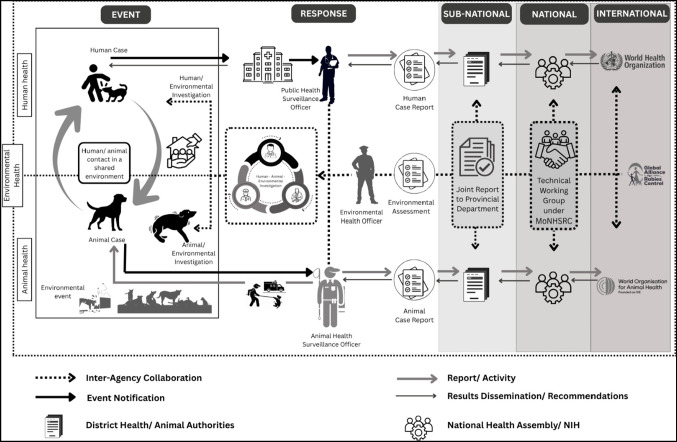
Analytical framework showing operationalization of the One Health Approach for Rabies Elimination in Pakistan. [16, 23–25]

A comprehensive analytical framework, grounded in the One Health approach and adapted from prior studies [16, 23–25], was used to examine the institutional and operational dynamics of rabies control in Pakistan (see Fig. 2). The framework outlines three interlinked domains that are essential to rabies prevention and response:

1. Human health services – encompassing post-exposure prophylaxis, wound care, policy formulation, and community awareness.
2. Animal health services – including mass dog vaccination, stray dog population management, and veterinary response capacity.
3. Environmental health services – particularly waste management and urban sanitation, which influence stray dog density and disease spread.

These domains are not standalone; their effectiveness depends on the structured intersectoral coordination at community, sub-national and national levels. The framework emphasizes that cross-sectoral collaboration − such as timely case reporting, data sharing and joint response planning − is critical for managing dog bite incidents and preventing outbreaks.

Figure 2 also identify enabling systems that support or hinder coordination. Legal mechanism (e.g., municipal bylaws, bite notification systems), economic investments (e.g., funding for rabies vaccines, TNVR operations), and sociocultural factors (e.g., local perceptions of dogs, healthcare-seeking behaviors) are integrated inro each sectoral interface.

Together, these components reflect the real-world dynamics of rabies control in Pakistan, where siloed programs have historically limited progress. By mapping these interconnections, the framework highlights how aligned policy, operational readiness, and social behavior must converge for One Health to be effective.

This framework guided the development of both survey and interview tools and informed the coding structure used in qualitative analysis. It was particularly useful in assessing institutional roles, coordinated gaps, and opportunities for joint action.

Figure 2 illustrates how operational actions (e.g., dog bite response, vaccination, sanitation improvements) intersect with cross-sectoral support systems to shape rabies outcomes. It visualizes One Health not as a concept, but as a functioning model of interdependent action.

### Study Design & Setting

A convergent mixed-methods approach was used, combining quantitative data to assess behavioral, systemic, and institutional factors shaping rabies control in an urban Pakistani context. The study was conducted in Karachi, a megacity city with a high burden of dog-bite injuries, a complex governance structure, and ongoing TNVR pilot campaigns. Karachi was selected due to its epidemiological relevance and logistical feasibility.

**Fig. 3:**
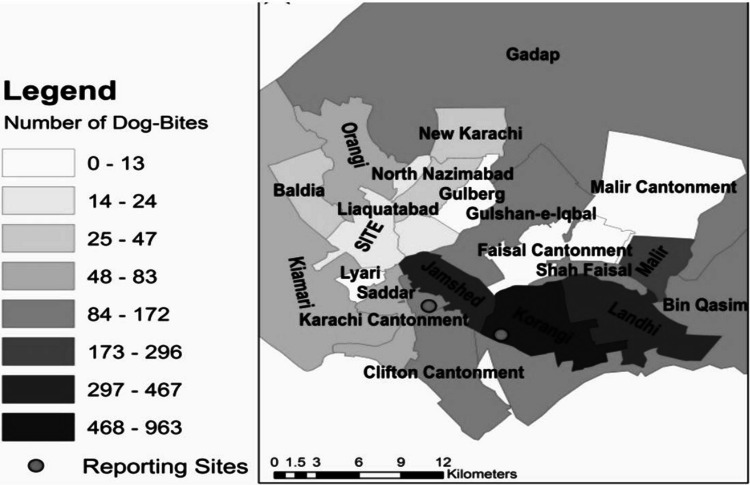
Spatial mapping showing the number of dog bite cases in Karachi from 2009–2011 [26]

For the quantitative component, two high-risk towns – Korangi and Bin Qasim- were randomly selected from a list of towns previously identified as rabies hotspots. Zaid et al. reported elevated incidence in Korangi, Malir, Bin Qasim, Jamshed, and Gadap town (see Fig. 3 for the spatial distribution of dog bite cases in Karachi)., while Ali et al. later confirmed the highest reported dog bites from Malir, followed by Korangi and Landhi [6, 26]. These towns were selected based on the previously reported high incidence of dog-bite cases: 468–963 in Korangi and 84:83 in Bin Qasim [26].

A cross-sectional KAP survey was conducted among community members in the selected towns. Concurrently, semi-structured interviews were held with key institutional stakeholders from the human, animal, and environmental sectors at district, provincial, and national levels. Both data strands were collected simultaneously, with equal emphasis, to allow for triangulated interpretation.

Qualitative and quantitative findings were triangulated during the interpretation stage, highlighting areas of convergence and divergence between stakeholder perspectives and community survey data. This approach aligns with the One Health framework, which emphasizes multisectoral coordination between community and institutional stakeholders.

### Study Participants

The study involved two distinct participant groups: community members and institutional stakeholders.

For the quantitative component, adult residents (aged > 18 years) of Korangi and Bin Qasim towns in Karachi were eligible to participate in the KAP survey. Inclusion criteria required participants to be permanent residents of the selected towns, with no professional association with human health, veterinary, or municipal institutions, and able to provide informed consent. Participants were selected using a systematic random sampling method from households in the identified areas.

For the qualitative component, the participants were selected on the basis of their institutional involvement and operational roles in the health system according to the One Health systems mapping approach [25]. Stakeholders were purposively recruited from human, animal, and environmental health sectors to reflect multisectoral perspectives critical for rabies control. These included individuals affiliated with public health institutions, veterinary departments, municipal governance, and civil society organizations with mandates relevant to zoonotic disease prevention.

### Sample Size and Sampling Technique

The sample size for the household survey was calculated using a single population proportion formula in OpenEPI, based on two parameters drawn from previous studies in Pakistan: a knowledge proportion (p) of 71% and a practice proportion of 47% [9, 27, 28]. Using a 95% confidence interval (Z = 1.96), a 5% margin of error (d = 0.05), and an assumed population of 17 million for Karachi, the estimated sample sizes were 317 for knowledge and 383 for practices. To ensure adequate statistical power and representation, the larger of the two estimates was selected, resulting in a final sample of 385 households.

Households were selected through systematic random sampling within two high-incidence towns- Korangi and Bin Qasim. Within each town, lists of residential blocks were used to define sampling intervals, and one eligible adult (aged > 18 years) per household was selected for participation using a random start and fixed skip pattern.

For the qualitative component, one stakeholder from all the 14 departments across human, animal, and environmental sectors was identified through purposive sampling based on their roles identified in a previous mapping exercise [25].

1. Human health professional (Karachi).
2. Animal health professional (Karachi).
3. Human Health Educator (Karachi).
4. Animal Health Educator (Karachi).
5. District Health Authority (Karachi).
6. District Animal Authority (Karachi).
7. District Municipal Corporation (DMC), Karachi.
8. Karachi Metropolitan Corporation (KMC).
9. Sindh Provincial Department of Health (DoH).
10. Sindh Institute of Animal Health (Karachi).
11. National Institute of Health (NIH).
12. Ministry of National Food Security and Research (MoNFSR).
13. Ministry of National Health Services Regulations & Coordination (MoNHSRC).
14. Expanded Program on Immunization, Sindh (EPI).

### Data Collection

For the quantitative component, data was collected using a structured questionnaire. The tool was adapted from previously validated KAP instruments used in Pakistan [9], and Ethiopia [29], and tailored to reflect the urban public health context of Karachi. Additional modules were added to assess environmental determinants – such as waste disposal practices and sanitation—and community awareness of intersectoral coordination concepts aligned with the One Health paradigm. The finalized questionnaire covered four key domains: household demographics, rabies knowledge, preventive practices, and environmental conditions (see Table 1). It was pilot-tested for clarity, comprehension, and contextual appropriateness.

**Table 1:** Survey domains and key indicators

Domain	Indicators
Household Demographics	Age, Gender, marital status, education, occupation, income, household size, children, pet ownership, dog-bite history
Rabies Knowledge	Awareness of rabies, transmission routes, signs/symptoms in humans/animals, affected species, and prevention
Preventable Practices	Interaction with free-roaming dogs, dog-population control preferences, PEP behavior, rabies vaccination status
Environmental Factors	Waste disposal, neighborhood sanitation, perceived links between environment and dog presence

For the qualitative component, semi-structured interview guides were developed for stakeholders spanning the human, animal, and environmental health sectors. The guides were informed by the One Health Systems Mapping and Analysis Resource Toolkit (OH-SMART) framework and insights from the OH-SMART workshop held in Pakistan [25]. Further refinement was informed by recent literature on integrated One Health frameworks [30].

The interview guides explored themes including intersectoral coordination, policy formulation and implementation, resource availability, diagnostic capabilities, and multisectoral mandates. All questions were open-ended and aligned with the study’s conceptual framework. The guide was reviewed by senior public health researchers and piloted to ensure thematic relevance.

Interviews were conducted in Urdu or English depending on participant preference. All interviews were audio-recorded with informed consent and detailed field notes were maintained. The sample size was guided by the principle of data saturation—interviews continued until no new themes emerged.

### Data Analysis

Quantitative data analysis was performed using IBM SPSS Statistics 21, focusing on descriptive summary statistics relevant to urban rabies epidemiology. Composite scores were generated for the knowledge (13 items) and practices (6 items) domains. Responses were scored as follows: 1 point for a correct answer, 0.5 for a partially correct answer, and 0 for an incorrect or “don’t know” response. Descriptive statistics—including means, standard deviations, and frequency distributions- were used to summarize respondent performance across domains. Normality of the knowledge and practice score distributions was assessed using the Shapiro–Wilk test to guide the appropriate interpretation of findings. No inferential statistics were applied due to the study’s primarily descriptive intent and the non-parametric distribution of some variables.

The qualitative interviews were audio-recorded, transcribed verbatim, and translated into English where necessary. Thematic analysis was conducted using Atlas.ti, applying a deductive coding approach guided by the study’s conceptual framework (see Fig. 2). Codes were refined iteratively during team debriefings to maintain analytical consistency and contextual relevance.

Triangulation occurred during the interpretation stage, where findings from both data strands were compared to identify convergences and divergences between community-level perceptions and institutional realities. This integration enhanced internal validity and provided a system-level understanding of rabies control dynamics under the One Health paradigm.

## Results

### Quantitative Results—KAP Survey

#### Participants’ Characteristics

**Fig. 4:**
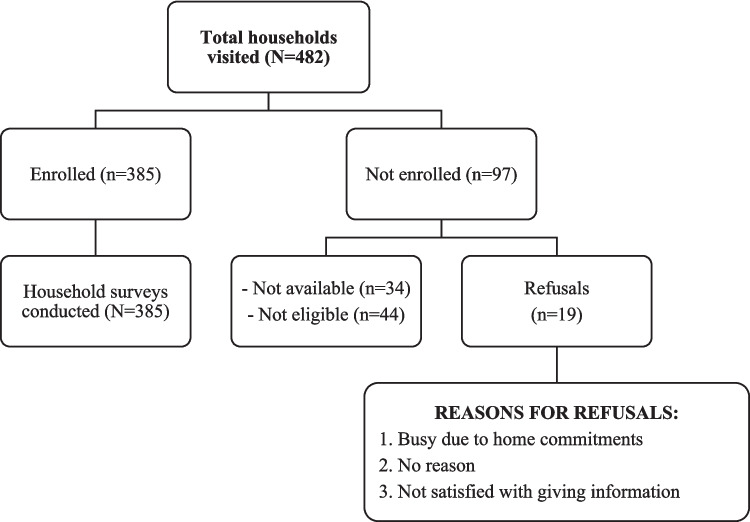
Total numbers of households visited & enrolled

A total of 482 households were approached, of which 385 eligible participants completed the questionnaire- 194 from Korangi and 191 from Bin Qasim towns (see Fig. 4). The sociodemographic characteristics of the participants are shown in Table 2. The majority of the respondents were women (75.1%, *n* = 289), while men constituted 24.9% (*n* = 96). The average participant age was 35.78 years (SD ± 12.5), with 81.8% falling between the ages of 19 and 45. Most respondents (88.6%) were married, and nearly half (49.3%) had no formal education. Secondary or higher education was reported to be 24.2%, while 26.5% had completed their formal education.

**Table 2:** Demographic profile of Survey Respondents in high-risk Karachi towns (*n* = 385)

Characteristics	*n (%)*
Sex	
Male	96 (24.9)
Female	289 (75.1)
Age (years)	
19–45	315 (81.8)
Above 45 years	70 (18.2)
Marital Status	
Married	341 (88.6)
Unmarried	44 (11.4)
Education Status	
No formal education	190 (49.3)
Secondary Education	93 (24.2)
Formal Education	102 (26.5)
Occupation	
Unemployed/Homemaker	239 (62.1)
Private/Government Employee	140 (36.3)
Student	6 (1.6)
Monthly Household Income	
≤ 40,000 PKR (230 USD)	350 (90.9)
> 40,000 PKR	6 (1.6)
Not disclosed	29 (7.5)
Household Size	
≤ 1–5 members	157 (40.8)
> 5 members	228 (59.2)
Children in Household	
No children	81 (21.0)
1–2 children	162 (42.1)
> 2 children	142 (36.9)
Family Exposure to Dog Bite	
Yes	137 (35.6)
- Children < 15 Years	**-** 51 (37.3 out of 137)
- Age 16–30 Years	**-** 43 (31.4 out of 137)
- > 30 Years	**-** 41 (29.9 out of 137)
No	248 (64.4)
Pet Ownership	54 (14.0)
Rabies Vaccination in Family	117 (30.4)

Regarding employment, 62.1% were either unemployed or homemakers, and 32.2% were engaged in private sector jobs. Government employees and students made up a small fraction of the sample (4.1% and 1.6% respectively). Household monthly income was under PKR 40,000 for 90.9% of respondents, though 7.5% declined to report their earnings. The average household size exceeded five members in 59.2% of cases.

Dog bite exposure within the family was reported by 35.6% of participants (*n* = 137). Among these, 37.3% of bite victims were children under 15, while 31.4% were aged 16–30, and 29.9% were over 30. Only 14% of households owned a pet, and 30.4% of families reported having at least one member who had received rabies vaccination.

#### Knowledge Scores

The mean knowledge score among respondents was 5.54(SD ± 2.43) out of 13. Only 21.3% scored 8 or above, and nearly half (47.5%) scored below 6, reflecting substantial knowledge gaps (see Table 3).

**Table 3:** Knowledge regarding rabies among respondents in high-risk towns, Karachi (*n* = 385)

Knowledge items	Frequency (*N*)	Percentage (%)
1. Ever heard about Rabies		
Yes	190	49.4
No	195	50.6
If yes, source of information? (n = 190)		
From someone	161	84.7
Mass media	13	6.9
Both	16	8.4
2. Potential Rabies Reservoir		
Dogs/Cats/Bats	170	44.16
Dog, cats with other animals	28	7.27
Other animals	6	1.56
Don’t know	181	47.01
3. Are stray dogs dangerous?		
Yes	333	84.5
No	36	9.3
Don’t know	16	4.2
4. Is Rabies a problem in Pakistan?		
Yes	263	68.3
No	15	3.9
Don’t know	107	27.8
5. Cause of Rabies		
Virus	47	12.2
Bacteria	13	3.4
Psychological problem	52	13.5
Don’t know	268	69.6
Bad hygiene	6	1.3
6. Severity of Rabies		
Not severe	5	1.3
Severe but recovery possible	145	37.7
Severe resulting in death	85	22.1
Don’t know	150	38.9
7. Can rabies be controlled?		
Yes	244	63.4
No	10	2.6
Don’t know	131	34
8. How is rabies transmitted from animal to human?		
If an animal bites or scratches, you	202	52.47
If an animal bites or plays with you	44	11.43
If an animal plays or sits near you	24	6.23
Don’t know	115	29.87
9. How to prevent dog bites?		
Educate people & keep a safe distance	105	27.27
Dog culling	145	37.66
Both educate and cull dogs	4	1.04
Cannot be prevented	68	17.66
Don’t know	63	16.36
10. Signs of a rabid dog		
Excessive salivation, aggression, barks a lot	139	36.1
Salivation, plays with everyone	71	18.44
Eats more, maggot wound, plays	94	24.42
Don’t know	81	21.04
11. What are the clinical signs of rabies in humans?		
Biting, behavior change, hydrophobia, fever	35	9.1
Madness, barks, stops eating/drinking	45	11.69
Madness	111	28.82
Don’t know	194	50.39
12. When to get rabies vaccine after bite?		
Immediately	321	83.4
After 2 or 3 weeks	3	0.8
At any time	0	0.0
Don’t know	61	15.8
13. Can vaccinating dogs prevent rabies outbreak?		
Yes	200	51.9
No	25	6.5
Don’t know	160	41.6

Item-wise analysis revealed that although 49.4% of participants had previously heard of rabies, awareness of the disease’s etiology and transmission was limited. Only 12.2% correctly identified a virus as the cause, 44.2% knew about animal reservoirs, and 22.1% were aware of the disease’s severity. About 52.5% accurately reported that rabies is transmitted through bites or scratches. Recognition of clinical signs and symptoms in animals was noted in 36.1% of the participants, while awareness of human rabies symptoms was even lower at 9.1%. A majority (83.4%) recognized the importance of prompt vaccination following a dog bite, and 51.9% believed that dog vaccination could help prevent rabies outbreaks.

These findings highlight both limited overall knowledge and specific misconceptions in the surveyed communities, underscoring the need for enhanced public education and targeted health messaging.

#### Practices Regarding Rabies Prevention

The total practice score ranged from 0–6, with a mean of 3.86 (SD ± 1.17) and a median of 4, indicating moderate adherence to recommended rabies prevention and response behaviors. Approximately 28.8% of respondents achieved high practice scores (≥ 5), while 15.3% demonstrated low scores (< 3), highlighting substantial variability in preventive practices (See Table 4).

**Table 4:** Practices regarding rabies and free-roaming dogs among community respondents in high-risk towns, Karachi (*n* = 385)

Practice Items	Frequency (*n*)	Percentage (%)
1. What should be done to control rabies in animals?		
Proper TNVR programs	152	39.5
Through TNVR & dog culling	9	2.3
Dog culling	109	28.3
Cannot be controlled	24	6.2
Don’t know	91	23.6
Have you or anyone you know ever been vaccinated against rabies?		
Yes	117	30.4
No	268	69.6
If yes, how many injections were received?		
1–5 injections	53	-
More than 5 injections	47	-
Don’t remember	17	-
2. What should be done if bitten by a dog?		
Wash wounds with soap and water/visit hospital	239	62.1
Wash the wound and use remedies	60	15.6
Religious, spiritual, or home remedies	49	12,7
Let the wound heal itself	0	0.0
Don’t know	37	9.6
3. What treatment should be given if get exposed to rabies?		
Antibiotics & Tetanus injection	5	1.3
Vaccine or Injection into the wound	292	75.8
Don’t know	82	21.3
Injection into the stomach	5	1.3
Injection into the wound & stomach	1	0.3
4. When would you visit hospital if bitten by a dog?		
Immediately after the bite	370	96.1
A day after the bite	1	0.3
2–14 days after a bite	0	0.0
Don’t know	14	3.6
5. What would you do if you saw a suspicious (rabid) dog in your area?		
Report to municipality	156	40.5
Poison or shoot it	154	40.0
Do nothing and keep a safe distance	68	17.7
Don’t know	7	1.8
6. Where do you normally dispose of your household litter?		
To the sanitary worker	130	33.8%
Near your house	160	41.6%
Away from your house	81	21%
In an empty plot	14	3.6%

Item-wise, 39.5% of respondents were aware of the need for TNVR programs to control rabies in animal populations. Around 62.1% reported that washing the wound with soap and water, along with visiting a healthcare facility after a dog bite, was essential. Most participants (75.8%) recognized the necessity of vaccination or injection after rabies exposure, and 96.1% stated that hospital visits should occur immediately following a dog bite. Only 40.5% indicated that they would report suspected rabid dogs to the municipality. In terms of environmental practices, 33.8% reported correct waste disposal, while the remainder disposed of their waste in open plots or areas near their homes, potentially sustaining stray dog populations.

These findings suggest that, despite reasonable awareness of some recommended practices, significant gaps remain in community engagement with effective rabies control measures.

## Qualitative Results

### Overview of Stakeholder Participants

For the qualitative component, 14 stakeholders were initially targeted from sectors integral to rabies control in Karachi, Pakistan, including human health, animal health, and environmental management (see Table 5). Of these, 10 were successfully enrolled and interviewed; the remaining four could not be reached or declined participation due to institutional or logistical barriers.

**Table 5:** Stakeholders targeted and interviewed

Sectors	Enrolled (*n* = 10)	Not enrolled (*n* = 4)
Human Health	• Doctor (Infectious Disease, Karachi) • Human Health Educator (Rabies Project Director, Karachi) • District Health Officer, Central, Karachi • EPI, (Additional Director General, Sindh) • National Institute of Health (Executive Director)	• Sindh Provincial Department of Health • Ministry of National Health Services Regulation & Coordination
Animal Health	• Veterinary (Karachi) • Animal Health Educator (Rabies Prevention Clinic, Karachi) • Sindh Institute of Animal Health (Director General)	• District Animal Authority, Sindh (does not exist in Karachi) • Ministry of National Food Security and Research
Environmental/Municipal	• Karachi Metropolitan Corporation (Municipal Commissioner) • District Municipal Corporation (Municipal Commissioner, Central)	-

The enrolled participants included policymakers, Program directors, field supervisors, municipal authorities, and technical experts, all relevant to rabies surveillance, animal vaccination, public health outreach, or waste management initiatives, identified by previous mapping exercise [30]. The diversity of this sample allowed for a comprehensive examination of systemic and operational barriers as well as potential enablers for rabies control from a One Health perspective.

### Thematic Findings

#### Barriers to Implementing One Health for Rabies Control

Stakeholders revealed significant barriers to implementing rabies control strategies in Pakistan, spanning knowledge gaps, resource constraints, coordination challenges among sectors, policy misalignment, and environmental factors. The following themes were identified using a deductive approach aligned with the study’s One Health conceptual framework.

**4.2.1.1. Knowledge and Awareness Gaps**

There was a strong sentiment that One Health principles are not yet widely understood or applied within local government and health sectors. When asked about One Health awareness, several stakeholders mentioned:

*“No, I have never heard about One Health. Maybe I have heard it but don’t remember what it is about.”*

Sindh Rabies Control Program, KMC

*“I have never heard about one health approach in health.”*

DHO, Karachi

*“One health approach is something general, there is a general perspective that health is very important for any human being. Whether you live in any country, only a healthy human can move forward in a society so that human needs to consult with the doctor, whether it is a child or an adult.”*

District Municipal Corporation, Karachi

Stakeholders also highlighted a significant knowledge gap within the community regarding health-seeking behavior and preventive measures, which include the use of outdated and ineffective home remedies.

*“People get bitten, and they use home remedies. They don't go to the hospital to seek medical care and meanwhile, the wound is healed but after some time, the patient develops rabies.”*

Human Health Educator

**4.2.1.2. Systemic Challenges**

Our analysis revealed that legal ambiguities, economic constraints, and social norms significantly influence the operationalization of One Health strategies for rabies control in Karachi.

**a. Intersectoral Coordination**

The current system lacks effective interdepartmental communication and collaboration, leading to inefficiencies in addressing rabies control. Various departments do not routinely coordinate, resulting in disjointed efforts and missed opportunities for integrated management.

*“There is no proper coordination between animal, human, and environmental departments right now, but there should be.”*

DHO, Karachi

Moreover, it was found that there is no formal body in Pakistan that ensures integration. This absence of a dedicated forum for One Health significantly hinders cohesive action.

*“There is no forum that is available within the government or Pakistan that ensures that there is one health concept in real.”*

Sindh Institute of Animal Health

*“The Ministry of National Health Service and Regulation is not a ministry for interdepartmental coordination. It is more for human health and related matters.”*

NIH, Pakistan

**b. Resource and Funding Limitation**

**i. Resource Availability**

Resource limitations have emerged as a critical challenge. Many stakeholders voiced concerns about the reliance on imported vaccines, with no local production to support a sustainable response.

*“The vaccine and immunoglobins are also imported from different countries, which makes it costly.”*

EPI

*“Vaccine production was one of the mandates when NIH was formed, and given that rank, we produced some of the vaccines. However, 5 years earlier they were all closed.”*

NIH

Furthermore, diagnostic capabilities were also found to be lacking, with healthcare providers relying on clinical symptoms rather than confirmatory tests.

*“We don’t have any test in Pakistan, so we only diagnose rabies with clinical signs and symptoms and with the association of a dog bite history.”*

Human Health Educator.

**ii. Resource Allocation**

Inadequate resource allocation was also highlighted as a significant barrier. Legal and bureaucratic challenges further complicate the efficient distribution of resources, leading to critical gaps in rabies control efforts.

*“Because of the court issue, the rabies project was given to the local government. Currently, there are no experts in the local government, neither the veterinary nor the human health. Therefore, they can’t implement this project, and we are now hanging in the middle.”*

Sindh Institute of Animal Health

Despite the shortage throughout the country, some departments mentioned the availability of unused vaccines which highlights the inequitable distribution of resources.

*“We have an enormous stock of rabies vaccinations, which has never been even used once. The stock that we have is now getting expired.”*

DHO, KHI

**iii. Funding Limitations**

Negligible funding directed toward animal health and preventive strategies exacerbates these challenges. The low priority given to animal health in budget allocation restricts the ability for proactive rabies prevention.

*“In the animal source, you can control rabies with the Rs.10 cost but when it crosses the species barrier and comes to humans. It’s a thousand times dangerous and will cost you large.”*

Sindh Institute of Animal Health

**4.2.1.3. Surveillance and Integrated Bite Management**

A striking finding was the absence of a cohesive surveillance system and registry for rabies in Pakistan. Therefore, rabies remains a non-notifiable disease, leading to underreporting and challenges in assessing the true disease burden.

*“Unfortunately, we do not have any surveillance, but there is a very vague estimate of 2000-5000 human rabies deaths annually. I believe this is just the tip of the iceberg, as we see more than 1000 dog bite cases every month in this hospital.”*

Human Health Educator, Karachi

*“There is no registry; this was just a vertical program coming under the Pakistan Health Research Institute.”*

NIH, Pakistan

Moreover, the absence of established protocols and integrated management systems was evident.

*“In all over the world, when you see a rise in dog bite cases, you go by a protocol and inform the other stakeholders, but unfortunately it is not done here in Pakistan.”*

Human Health Educator

**a. Vague Role Definition**

There is no consensus on roles and strategies among various departments, which leads to inefficiencies and delays in response to rabies outbreaks. Stakeholders indicated a lack of clarity and consensus on the roles and responsibilities of different departments involved in rabies control, complicating effective response strategies.

*“The only responsibility that I have would be to provide a vaccine to the affected individual; reporting and handling of the dog case is not our responsibility.”*

DHO, Karachi

“*The hospitals also have a big role in this other than treating the patients, many times there comes a phone call that there was a dog bite in a certain area. So that means you just found out that in that area there are so many stray dogs, you need to tell someone”.*

NIH

**4.2.1.4. Environmental Factors**

**a. Improper Waste Management**

Improper waste disposal is a significant concern that indirectly contributes to the rabies problem by increasing dog populations. This issue highlights the One Health approach, where environmental health directly affects animal and human health.

*“Waste management is a big issue in Pakistan because dogs feed on that. Therefore, this affects the population as well as transmits diseases in many ways.”*

Animal Health Professional

*“There are penalties in the law, the implementation is the issue.”*

KMC

**4.2.1.5. Rabies Management and Policy**

**a. Mass Dog Vaccination and Population Control**

On the animal side, the absence of a structured body for rabies control due to a lack of expertise was found to be a major barrier to effective mass dog vaccination and population control strategies. The challenges include the lack of a specific facility to cater to small urban animals, annual vaccination, lack of finances, etc.

*“The government does not have any expertise in animal health. The livestock department we have is very specific. We have wildlife, which is a separate department. Next, we have the livestock department, which is completely different. The third group includes urban animals, and the main ones are donkeys, dogs, and cats. There is no department for these urban animals.”*

DMC, KHI

*“Annual dog vaccination is very challenging, and we have experienced that. We have seen running after dogs, catching them, it's a huge physical work and it requires proper training, proper planning, and properly designed work”.*

Human Health Educator, Rabies Free Pakistan

*“The problem is that we need a lot of money and financial resources to combat that; currently, we do not have that.”*

KMC, KHI

**b. Incoherent Policies and Strategic Misalignment**

There is still no national rabies program, but a public program was launched in May 2022 by the local government department, Sindh, which was named the “Rabies Control Program Sindh” [31]. However, unfortunately, it is also not formulated on the principles of One Health. There is a disproportionate focus on human health, neglecting crucial aspects such as animal population control and environmental management, which leads to ineffective management overall.

*“Sindh Rabies Control Program is focusing on human health more rather than controlling the dogs and other resources so therefore it is not moving in the right direction currently.”*

Sindh Institute of Animal Health

*“Sindh Rabies Control Program is still in the court matters with the local government."*

Sindh Institute of Animal Health

*“As a country, we do not see a big plan there we do not see a National Rabies program.”*

Human Health Educator, Karachi

**c. Divergent Views on Policy Needs**

There is a lack of consensus among stakeholders on the necessity and scope of rabies-related policies, which reflects differing views on strategic approaches. Some stakeholders question the need for a national policy, whereas others emphasize the importance of political will and expert involvement in addressing rabies effectively.

*“I don’t think we need a national policy for rabies! There is always an alternative solution.”*

KMC, Karachi

*“Dog culling has not shown its effectiveness because it is still not properly implemented by KMC & DMC as it should be. Additionally, it has been decreased now.”*

DHO, Karachi

*“First of all, you know the political will should be there. Once the will is there, then it is not that big of a problem.”*

Human Health Educator, Karachi

#### Facilitators for Achieving One Health Implementation in Rabies Control

The following themes were identified using a deductive coding approach guided by the study’s One Health conceptual framework, and reflect operational, policy, and community-level enablers described by stakeholders.

**4.2.2.1 Collaborative Frameworks and Multi-Sector Integration**

Stakeholders frequently cited the existence of collaborative platforms for One Health, particularly in Antimicrobial Resistance (AMR), as models for rabies control. These multisectoral frameworks facilitate coordinated action and data sharing across human, animal, and environmental health domains.

*“One platform that is on One Health is AMR, which is centrally operated from Islamabad. Veterinary and human health experts combine, and they have a common platform where they can share their information. We now have a common reporting system as well. A similar forum can also be utilized for rabies as well.”*

Sindh Institute of Animal Health

*“Rabies should be eliminated through a proper channel and single-person effort is not good enough. Different programs are running but if we combine them all in one program, it will be better.”*

Animal Health Professional

**4.2.2.2. Innovative Approaches to Rabies Control**

**a. Small-Scale Programs and Campaigns**

Stakeholders recognized the positive impact of small-scale programs such as Rabies Free Pakistan, which have demonstrated success in TNVR implementation and public awareness. These programs serve as proof-of-concept for scalable One Health strategies adapted to local needs.

*“The Sindh government has taken a great initiative itself called the Sindh Rabies Control Program; we look forward to it.”*

Animal Health Educator

*“We are a rabies prevention center in Indus Hospital; at the same time, we are a training center for the entire province and other cities. Throughout the year, we do several things such as awareness campaigns in the communities where we get more dog bite cases.”*

Human Health Educator

**b. Adoption of Technology**

The integration of electronic data systems in hospitals has improved case tracking, follow-up, and monitoring- demonstrating the utility of technology in rabies surveillance and research.

*“We are a paperless hospital, and all the data and patient-related information are documented in an electronic database… The data that we keep doesn’t just benefit the patients but also for various research”.*

Human Health Educator

**4.2.2.3. Policy Initiatives and Legal Frameworks**

Stakeholders highlighted recent policy approvals and pilot programs in Sindh and Punjab as signs of growing political will for coordinated rabies control. The development of Standard Operating Procedures (SOPs) and formal policies was noted as an important step toward national scale-up and legal backing for One Health approaches.

*“The cabinet approved rabies policy in Punjab. SOPs were subsequently used to handle dogs and control rabies transmission. They also published the data on it.”*

Sindh Institute of Animal Health

*“These 75 years have taught us that we stand nowhere when it comes to the results that we expected by dog culling. However, fortunately, for the last few years, I have been seeing that many people are trying to opt for a better solution, i.e., TNVR. Sindh is the first province in Pakistan to start this program to rule it out, and let’s see how it goes!”*

Rabies Control Program, Sindh

National stakeholders also emphasized the need to incorporate preventive measures into policies and actions for better control of rabies, highlighting the value of “upstream” strategies targeting animal reservoirs and environmental contributors.

*“A multifaceted approach is needed. It is not only that the patient comes in, once the dog bites a person then the treatment is started. The thing is that we should start from the preventive side, the most important thing is that you try to stop the breeding of stray dogs”.*

NIH, Pakistan

**4.2.2.4. Mass Dog Vaccination and Population Control Initiatives**

The willingness of stakeholders to scale up TNVR and systematic dog vaccination was viewed as a critical facilitator for sustainable rabies control, directly aligning with One Health’s operational principles.

*“It will take us one to two years to make the rabies vaccine for dogs available. Once we have the vaccines, then we are going to vaccinate dogs regularly and control their population.”*

Sindh Institute of Animal Health

*“We have already provided the list of 68 hospitals in Sindh that can be utilized for TNVR treatments and surgeries to the local government.”*

Sindh Institute of Animal Health

**4.2.2.5. Effective Waste Management and Environmental Control**

Stakeholders acknowledged that municipal and community action on waste management is essential for breaking the animal-human transmission cycle.

*“The dog population is multiplying so fast... The reason behind this is because they get leftover food from the garbage on which they thrive!”*

Animal Health Educator

*“Citizens have the responsibility to be mindful of how they are throwing away the trash and then obviously municipalities need to know different strategies that they can implement. We do not have good recycling mechanisms here in Pakistan. This requires the involvement of multiple stakeholders who come together and solve this problem of waste disposal.”*

Human Health Professional

*“Without the penalties and law enforcement, nothing can be done. We are not accustomed to working without pressure from the authorities above.”*

DMC, Karachi

**4.2.2.6. Resource Mobilization and Strategic Deployment**

Ongoing efforts to secure funding, build vaccine production capacity, and improve workforce skills were seen as necessary enablers for sustainable rabies control.

*“The government should allot a budget to this sector also. Once there is political will, then we can do anything.”*

EPI

*“There is an enormous pressure on the NIH, so we will definitely try to rejuvenate and hopefully we will start making it in the future.”*

NIH

**4.2.2.7. Community Involvement and Educational Outreach**

Community-based education and engagement, including leveraging media campaigns and integrating rabies messaging into existing outreach programs, were cited as essential facilitators.

*“Awareness campaigns should target the veterinary field experts, local government officials, dog and pet owners, school authorities and the public health large through mass media.”*

Sindh Institute of Animal Health

*“We should engage everybody as it needs to be added to all levels of the pyramid. We can utilize already going programs such as the lady health worker program, where she is already going and imparting education about so many different things.”*

Human Health Educator, Karachi

*“We can go to schools and small towns and can also involve the education department, develop literature, and make cartoons.”*

Sindh Rabies Program

**4.2.2.8. Enhance Surveillance and Diagnostic Capabilities**

Stakeholders recognized the development of more robust, active surveillance systems- including hotlines and electronic reporting- as a key facilitator for early detection and integrated response.

*“It is better that rather than passive surveillance, there should be active surveillance. Put in a hotline where rabid dogs can be notified, make it public facing awareness and anybody from the public can call up.”*

Animal Health Educator

During the interviews, stakeholders mentioned the current operational surveillance system, highlighting its role in the community.

*“The Team of Field Epidemiology and Disease Surveillance Division (FEDSD) at the NIH is working to develop a proper rabies surveillance system.”*

Human Health Professional

*“We have been working on it. In fact, right now, the integrated Disease Surveillance and Response (IDSR) has already been launched, and it is functional. We use DHIS too which is a WHO-compatible software, and we will keep using that to collect all the data.”*

NIH, Pakistan

### Integration and Triangulation of Findings

The study used a convergent mixed-methods approach, integrating quantitative KAP survey data from community respondents with qualitative insights from institutional stakeholders. This triangulation provided a comprehensive perspective on barriers and facilitators to rabies control in Karachi within a One Health framework.

#### Points of Convergence

**4.3.1.1. Knowledge Gaps**

Both surveys and interviews consistently revealed major deficits in community knowledge and awareness regarding rabies transmission, prevention, and post-exposure management. Quantitative data showed that fewer than half of respondents recognized key preventive actions or risk factors, a finding echoed by stakeholders, who highlighted reliance on traditional remedies and limited understanding of the One Health approach.

**4.3.1.2. Systemic Barriers**

Quantitative evidence of suboptimal preventive practices was reinforced by qualitative accounts of fragmented intersectoral coordination, unclear role delineation, and insufficient resource allocation- barriers identified at both household and institutional levels.

**4.3.1.3. Environmental Risks**

Survey data indicated that only a minority practiced correct waste disposal, a result qualitatively validated by stakeholders who linked improper waste management to increased stray dog populations and high rabies risk.

#### Points of Divergence

**4.3.2.1 Policy and Operational Awareness**

While community-level respondents rarely mentioned the impact of policies or operational initiatives, stakeholders described a range of existing pilot programs, recent policy advances, and multisectoral platforms (such as the Rabies Control Program and AMR One Health models) that could be leveraged for rabies control.

**4.3.2.2. Facilitators and Innovations**

Stakeholder perspectives highlighted specific innovations- such as TNVR scale-up, digital surveillance systems, and intersectoral campaigns- and expressed optimism about ongoing progress. By contrast, community respondents did not report awareness of these activities, most likely due to the absence of such items in the questionnaire as well as limited direct engagement or communication about these innovations at the household level. It is possible that greater community engagement with these initiatives could enhance awareness and practice scores in future assessments.

## Discussion

This study systematically identified the practical enablers and barriers to rabies control strategies in Karachi, Pakistan through a One Health framework, integrating community (KAP survey), and cross-sectoral stakeholders (qualitative interviews). To our knowledge, this is the first mixed-methods study in Pakistan to triangulate household and institutional insights for rabies control.

A major finding of the study was the limited awareness of rabies among residents of high-risk towns, with a knowledge score of 42.62%, which was substantially lower than prior national estimates. This gap persisted despite ongoing exposure to rabies risk, underscoring the inadequacy of passive information transfer and the need for targeted, context-specific education in urban settings. Similar findings have been observed in other low-resource contexts [32–34].

In parallel, stakeholder interviews revealed limited understanding and operationalization of the One Health approach at the district and municipal levels. This aligns with global evidence calling for the integration of human, animal, and environmental health sectors to achieve effective rabies control [3, 35]. Addressing these knowledge and implementation gaps is essential for developing a coordinated national response.

Systemic barriers- especially the lack of structured intersectoral collaboration- were prominent. There is no formal mechanism for coordination across sectors at any administrative level. These challenges are not unique to Pakistan; similar issues exist in other South Asian countries implementing One Health strategies [3, 36]. Institutionalization of a centralized, multi-sectoral platform and leveraging digital tools for Integrated Bite Case Management (IBCM) could address this gap [37].

Political will and resource limitations were also critical. Government commitment to small animal health remains inadequate, and reliance on imported vaccines and limited diagnostics are certain challenges shared by other LMICs as well [38, 39]. Investment in local diagnostic and vaccine production capabilities is a priority for sustainable control.

The absence of a robust surveillance system was another key limitation. As rabies remains un-notifiable, underreporting persists, and the true disease burden is unclear. Other rabies-endemic countries have demonstrated the value of robust national surveillance for rabies elimination [40–43]. A reliable, integrated surveillance system is essential for Pakistan’s rabies control efforts.

Beyond operational and institutional challenges, our findings highlight that effective rabies control through One Health requires addressing legal, social, and economic factors alongside health sector coordination. Legal frameworks enable interdepartmental collaboration and enforcement; social determinants affect public engagement and behavior change; and economic investment shapes the sustainability and reach of rabies interventions. This expanded conceptualization is increasingly recognized in recent One Health literature [18, 44].

Despite these barriers, multiple facilitators emerged like the presence of existing models (such as Rabies Free Pakistan, Sindh Rabies Control Program, and AMR One Health platforms) which demonstrate institutional willingness and practical experience with cross-sectoral collaboration and TNVR implementation [12]. Similar efforts have been successfully implemented in other LMICs such as Bangladesh [21] and India [45, 46]. The willingness of all levels of the stakeholder pyramid to engage in One Health projects presents a great opportunity for scaling these initiatives.

Crucially, the integration of AMR platforms for One Health provides a ready template for cross-sectoral dialogue and data sharing in rabies management. The successful integration of One Health principles into India’s national rabies control program may provide an actionable model of such functional convergence [22]. The convergence of these experiences, with active government support, can accelerate progress toward national rabies elimination targets.

Moreover, the lack of an integrated bite management system and insufficient political prioritization require urgent action. Lessons from other LMICs- including Bhutan, Thailand, and Sri Lanka- underscore the need for a national plan, intersectoral collaboration, and adequate resource allocation to achieve effective rabies control [47]. The establishment of a central coordinating body, resourced and empowered, should be a priority for sustainable rabies management in Pakistan Beyond.

### Study Limitations

While this study integrated community and stakeholder perspectives using a robust mixed-method approach, certain limitations must be acknowledged. The stakeholder sample was restricted to individuals available and accessible in Karachi and online, which may restrict broader representativeness. Non-responsiveness from some departments created gaps in institutional participation, and qualitative findings are based on self-reported data which may be influenced by recall or reporting bias. Finally, the study focused on urban high-risk settings, and findings may not be fully generalizable to rural areas or other regions across Pakistan.

### Recommendations

The results of this mixed-methods study have several implications for policy and practice.

- The formation of a dedicated, well-resourced national rabies coordinating body is essential, with mandates for multisectoral coordination and centralized surveillance.
- Targeted education and sustained public awareness campaigns- especially in high-risk urban areas- are required to address knowledge and practice gaps.
- Investment in local vaccine and diagnostic production, along with cross-sectoral One Health training, will improve operational effectiveness.
- Building on successful models like the AMR One Health platforms and Rabies Free Pakistan can accelerate cross-sectoral integration and innovation.
- The adoption of electronic and active surveillance systems for rabies should be prioritized to ensure timely detection, data sharing, and response.

Overall, operationalizing these recommendations into practice in line with One Health principles − and ensuring that legal, social, and economic factors are addressed alongside technical interventions − will be essential for sustained rabies control in Pakistan. This approach can also serve as a model for other LMICs.

## Conclusion

The findings revealed critical knowledge, behavioral, and systemic coordination gaps for effective rabies control in Pakistan. However, it also highlighted practical opportunities for progress. By integrating the perspective of both affected communities and institutional stakeholders, the findings underscore the urgency of a unified national response- rooted in One Health principles, political will, and broader stakeholder engagement. The elimination of rabies in Pakistan will require coordinated policy action, intersectoral collaboration, local capacity building, and persistent public advocacy.

## Data Availability

The datasets generated and/or analysed during the current study are available from the corresponding author upon request.

## Abbreviations

AMR: Antimicrobial Resistance
DHO: District Health Officer
DMC: District Municipal Corporation
EPI: Expanded Program on Immunization
ERC: Ethical Review Committee
FEDSD: Field Epidemiology and Disease Surveillance Division
KAP: Knowledge, Attitude, and Practices
KHI: Karachi
KMC: Karachi Metropolitan Corporation
LMIC: Low- and Middle-Income Country
NIH: National Institute of Health
OH-SMART: One Health Systems Mapping and Analysis Resource Toolkit
PKR: Pakistani Rupee
SAARC: South Asian Association for Regional Cooperation
SOP: Standard Operating Procedure
TNVR: Trap-Neuter-Vaccinate-Return
USDA: United States Department of Agriculture
